# Maternal and fetal predictors of fetal viral load and death in third trimester, type 2 porcine reproductive and respiratory syndrome virus infected pregnant gilts

**DOI:** 10.1186/s13567-015-0251-7

**Published:** 2015-09-25

**Authors:** Andrea Ladinig, Carolyn Ashley, Susan E Detmer, Jamie M Wilkinson, Joan K Lunney, Graham Plastow, John CS Harding

**Affiliations:** Department of Large Animal Clinical Sciences, Western College of Veterinary Medicine, University of Saskatchewan, 52 Campus Drive, Saskatoon, SK S7N5B4 Canada; University Clinic for Swine, Department for Farm Animals and Veterinary Public Health, University of Veterinary Medicine Vienna, Veterinaerplatz 1, 1210 Vienna, Austria; Department of Veterinary Pathology, Western College of Veterinary Medicine, University of Saskatchewan, 52 Campus Drive, Saskatoon, SK S7N5B4 Canada; Department of Agricultural, Food, and Nutritional Science, Faculty of Agricultural, Life and Environmental Sciences, University of Alberta, 410 Agriculture/Forestry Centre, Edmonton, AB T6G2P5 Canada; Animal Parasitic Diseases Laboratory, Beltsville Agricultural Research Center, Agricultural Research Service, U.S. Department of Agriculture, 10300 Baltimore Blvd., Building 003, Beltsville, MD 20705 USA

## Abstract

Minimal research has focused on understanding mechanisms underlying porcine reproductive and respiratory syndrome virus (PRRSV) induced reproductive failure. We have completed a large-scale project investigating phenotypic and genotypic predictors of reproductive PRRS severity in which numerous clinical, pathological, immunologic and viral responses were characterized in dams and fetuses. The goal was to determine which phenotypic responses were associated with fetal viral load and death after experimental infection of pregnant gilts with type 2 PRRSV, thereby elucidating mechanisms of reproductive PRRS in third trimester pregnant gilts. The presence of fetal infection and increasing RNA concentration at the maternal-fetal interface were strong predictors of the probability of fetal death, while PRRSV RNA concentration in dam sera and systemic tissues were not associated with the odds of fetal death. Fetal infection and death clustered, indicating that the status of adjacent fetuses is crucial for lateral transmission and fetal outcome. Several systemic immune responses of gilts were associated with fetal outcome and viral load: interferon-α contributed to the probability of fetal death, but absolute numbers of T helper cells in early infection, absolute numbers of myeloid cells over time and interleukin 12 levels appeared protective. These results suggest specific immune responses may either contribute to, or protect against, transplacental virus transmission. The WUR10000125 SNP on chromosome 4, associated with PRRS resilience in nursery pigs, was not associated with reproductive outcome. Whereas past research suggested that fetal death results from events occurring at the maternal-fetal interface, we conclude that viral replication within fetuses and spread of PRRSV to adjacent fetuses are pivotal events in the pathogenesis of reproductive PRRS.

**Electronic supplementary material:**

The online version of this article (doi:10.1186/s13567-015-0251-7) contains supplementary material, which is available to authorized users.

## Introduction

In spite of PRRSV reproductive disease contributing millions in losses annually [1], a proportionately small amount of research has focused on the reproductive form of the disease. The outcome of infection in pregnant sows and gilts largely depends on the stage of gestation. PRRSV infection in early gestation can lead to embryonic infection and death, depending on the age of the conceptus [2-4]. Although porcine fetuses are susceptible to PRRSV at any stage of gestation upon direct intra-fetal or intra-amniotic inoculation, the virus does not readily cross the placenta in mid-gestation [5,6]. In contrast, PRRSV infection in late gestation consistently results in transplacental infection of fetuses and reproductive failure [6-9]. However, the exact mechanisms by which PRRSV transmits from the dam to her fetuses have yet to be determined. It is known that PRRSV replication in fetal implantation sites precedes fetal infection and induces apoptosis of infected and surrounding cells [10]. Furthermore, it has been suggested that the number of sialoadhesin positive (Sn^+^, CD169^+^)/CD163^+^ macrophages, the cells permissive to PRRSV, in the endometrium and placenta might be an important factor for transplacental virus passage [11]. Once the virus reaches the fetus it can be detected systemically in several fetal tissues including lung, liver, spleen, heart and kidney, but the virus is most consistently found in lymphatic tissues with the fetal thymus being proposed as the primary site of virus replication [7,12]. The absence of severe microscopic lesions in PRRSV infected fetal tissues suggests that fetal infection contributes little to the pathogenesis of fetal death [7,13,14].

We have recently completed the largest experimental pregnant gilt PRRSV inoculation study to date that has enabled the identification of phenotypic and genotypic factors associated with reproductive PRRS severity [15-19]. Numerous phenotypic responses were characterized, including clinical signs, virus levels in serum and tissues, changes in leukocyte subsets in gilt blood, cytokine protein levels over time in gilt serum and supernatants of stimulated peripheral blood mononuclear cells (PBMC), gross and microscopic pathology, fetal preservation and mortality [17-19]. For this experiment, dams were specifically selected from high and low birth weight (BW) litters in order to determine if BW of the dam influences PRRS severity. Additionally, genotyping for a major quantitative trait locus (QTL) on *Sus scrofa* chromosome (SSC) 4 conferring PRRSV resistance/susceptibility and explaining approximately 11 and 15% of the individual variation in weight gain and viral load, respectively, in experimentally infected nursery pigs [20-22] was performed on DNA from gilts, sires and non-autolyzed fetuses.

The specific objectives of the present study were to identify: 1) gilt level factors measured before inoculation that were associated with fetal mortality rate; 2) gilt and fetal level factors associated with increased or decreased odds of fetal death; 3) gilt and fetal level factors associated with PRRSV RNA concentration (viral load) in fetal thymus; and 4) gilt and fetal level factors associated with fetal preservation category. This is the first study to provide a quantitative estimate of the relative strength of association among various maternal and fetal factors and reproductive outcome following type 2 PRRSV challenge.

## Materials and methods

### Animal experiment and sample collection

The experiment was approved by the University of Saskatchewan’s Animal Research Ethics Board, and adhered to the Canadian Council on Animal Care guidelines for humane animal use (protocol #20110102). The experimental protocol is described in detail in Ladinig et al. [19]. Briefly, purebred Landrace gilts were selected over 12 bi-weekly replicates according to their birth weight. For this, the average litter BW of gilt piglets was compared to the historical averages of farm cohorts after controlling for total born litter size and parity. Fifty-six low and 58 high BW litters were identified as having a Z-score greater or less than 0.7, and one gilt with a BW closest to the average for the litter was selected from each [16]. Estrus was synchronized and gilts were bred homospermically to Yorkshire boars. On gestation day 85 ± 1 (0 day post infection (dpi)), 114 pregnant gilts were inoculated with PRRSV isolate NVSL 97–7895 (1 × 10^5^ TCID_50_ total; 2 mL intramuscularly and 1 mL into each nostril). Heparinized blood samples were collected at 0, 2, 6, and 19 dpi, and sera at 0, 2, 6, and 21 dpi. At 21 dpi, the gilts were humanely euthanized and necropsy examinations performed on gilts and their fetuses. Fetuses were numbered sequentially according to their position within each horn with “L1” and “R1” being the fetuses closest to the ovary on the left and right sides, respectively. Fetal preservation status was categorized as: viable (VIA), meconium stained (MEC), decomposed (DEC; dead with primarily white skin) and autolyzed (AUT; dead with over 50% brown discolored skin) [19]. The weight of fetuses and fetal organs, crown rump length (CRL) and sex of each fetus were recorded. From VIA and MEC fetuses, blood was collected from the axillary artery and serum subsequently separated and stored at −80 °C. Samples of lung, tonsil, reproductive (*Lnn. uterini*) and tracheobronchial lymph node were collected from each gilt. Samples of thymus and endometrium (including adherent fetal placental layers) adjacent to the umbilical stump were collected from each fetus. Tissue samples were immediately frozen at −80 °C until further processing or fixed in 10% formaldehyde enabling histological evaluation.

### Microscopic assessment of PRRSV-associated lesions

Gilt and fetal tissue samples collected in 10% buffered formalin were fixed for 24 h and routinely processed for Hematoxylin and Eosin (H&E) stained sections. Tissues were examined for the presence or absence of characteristic lesions associated with in-utero PRRS infection. These are primarily lymphohistiocytic endometritis or metritis and placentitis, but also included multicentric lymphohistiocytic arteritis, panvasculitis, and perivasculitis [13,14,23,24].

### Quantification of PRRSV RNA

PRRSV RNA concentrations were measured using a strain-specific, in-house quantitative reverse transcription polymerase chain reaction (qRT-PCR) [19] in gilt serum (0, 2, 6, 21 dpi), fetal serum from fetuses that survived until termination, and gilt and fetal tissues. Primers were designed to target a highly conserved region at the C-terminal end of ORF7 of NVSL 97–7895. Results were reported as logarithm base 10 target RNA concentration per mg or μL.

### Flow cytometric analyses of PBMC

Automated white blood cell (WBC) counts (Z2 Coulter Particle Count and Size Analyzer, Beckman Coulter Inc., FL, USA) and manual differential counts were performed (300 cells total) on heparinized blood samples collected at 0, 2, 6 and 19 dpi. PBMC were isolated by gradient centrifugation (Ficoll-Paque™ PLUS, GE Healthcare, Mississauga, ON, Canada) and analyzed phenotypically by flow cytometry (FCM) as described in detail [17]. Major PBMC populations were defined as follows: myeloid cells (CD172a^+^), natural killer (NK) cells (CD8α^+^CD3^−^), B cells (CD79α^+^), γδ T cells (T cell receptor γδ^+^), T helper cells (CD3^+^CD4^+^), and cytolytic T cells (CTLs) (CD3^+^CD8β^+^). Absolute numbers of different cell subsets were calculated using results from automated WBC and differential counts (total number of lymphocytes plus total number of monocytes).

### Cytokine testing

Gilt serum samples (0, 2, 6, 21 dpi) and supernatants from PBMC (0, 2, 6, 19 dpi) stimulated with either 10 ng/mL PMA (Sigma–Aldrich, Oakville, ON, Canada) and 250 ng/mL ionomycin (Sigma–Aldrich, Oakville, ON, Canada) (PMA/Iono) or with PRRSV isolate NVSL 97–7895 (multiplicity of infection = 1) were analyzed for innate, T helper 1 (Th1), T helper 2 (Th2), and regulatory cytokines/chemokines by Fluorescent Microsphere Immunoassays (FMIA) including interleukin (IL) 1β, IL4, IL8, IL10, IL12, chemokine ligand 2 (CCL2), and interferon alpha (IFNα) as described in detail [18]. An enzyme-linked immunosorbent assay (ELISA) was used to measure IFNγ levels in these samples as previously described [18].

### Determination of WUR10000125 alleles of gilts, sires and fetuses

Genomic DNA was extracted from 10–20 mg of fetal thymus using a DNeasy96 blood & tissue kit (Qiagen Inc., Toronto, ON, Canada) according to manufacturer’s instructions. High quality genomic DNA could not be obtained from AUT fetuses; therefore, only VIA, MEC and DEC fetuses were genotyped. Similarly, genomic DNA was extracted from 250 μL of boar semen, which was washed with 1000 μL of STE buffer and pelleted at 7000 × *g* for 5 min, or from 3–4 mm pieces of ear tissue from gilts. The semen pellet or tissue piece was then digested overnight in 180 μL of ATL buffer containing 20 μL of Proteinase K (Qiagen Inc., Toronto, ON, Canada), and was processed on the BioSprint (Qiagen Inc., Toronto, ON, Canada). The BioSprint is an automated instrument that uses magnetic beads to extract DNA. Once overnight digestion was complete, the BioSprint added 200 μL of AL Buffer (Qiagen Inc., Toronto, ON, Canada), 200 μL of Isopropanol, and 30 μL of MagAttract beads (Qiagen Inc., Toronto, ON, Canada). As per the manufacturer’s instructions, the extracted DNA was eluted in AE Buffer (Qiagen Inc., Toronto, ON, Canada).

The genotype of the WUR10000125 SNP was determined by a TaqMan assay (Forward primer: 5′ AGA CCT AGA ATC TCC ACA GAA TTT CCA 3′; Reverse primer: 5′ AAG TTA GAA TCT GCG CGA ATC GA 3′; Taqman MGB probe (VIC labeled): 5′ CTG GGT GAT AAA TAA AT 3′; Taqman MGB probe (FAM labeled): 5′ TGG GTGA TGA ATA AAT 3′; where the underlined bases are the A/G polymorphism scored). The G allele corresponds to the preferred allele (B) associated with higher growth and lower viral load in nursery pigs [20]. The TaqMan-MGB genotyping assay mix was supplied by Applied Biosystems (Burlington, ON, Canada). Genomic DNA was diluted to 10 ng/μL and 2 μL of DNA added to the DNA assay mix containing 5 μL of DNA TaqMan Universal PCR Master Mix, No AmpErase UNG (Applied Biosystems 4324018), 0.5 μL of TaqMan-MGB genotyping assay mix (20X), and 2.5 μL of water. After sealing the plate it was vortexed and centrifuged before PCR with thermocycler conditions: 60 °C for 30 s, 95 °C for 10 min, 95 °C for 15 s, 60 °C for 1 min, repeating steps 3–4 for 40 cycles and 60 °C for 30 s. The assay was run on a StepOne Plus (Life Technologies). Automatic allele calls were made and displayed as an allelic discrimination plot and confirmed by visual inspection against positive and negative controls.

### Statistical analyses

Fetuses were considered PRRSV positive by qRT-PCR if RNA was detected in either fetal thymus or fetal serum or both. The number of adjacent dead and PRRSV RNA positive fetuses was calculated by evaluating the dead-live status and PRRSV RNA status of the two adjacent fetuses to the left and right of each fetus (maximum 4). The relative position of each fetus within the horn was calculated by dividing the number of each fetus in the horn (range: 1 to 12) by the total number of fetuses within that horn. The presence or absence of histopathological lesions were analyzed as dichotomous outcomes. Similarly, the WUR10000125 allele of gilts and fetuses was dichotomized by combining the AG and GG groups, since the number of homozygous GG gilts and fetuses was low and it was previously demonstrated that the favorable G allele has dominance over the A allele [20,21]. Where required, the area under the curve (AUC0-19, AUC0-6) was calculated using the formula AUC = (t_1_-t_0_)(a_1_ + a_0_)/2 + (t_2_-t_1_)(a_1_ + a_2_)/2 + … + (t_n_-t_n-1_)(a_n-1_ + a_n_)/2.

All statistical analyses were performed using multilevel, mixed-effects regression models (Stata v13, StataCorp, College Station, TX). When developing full models, only variables that were highly significant (*P* < 0.1 or 0.05 depending on the model) in unconditional analyses, uncorrelated (Pearson’s correlation coefficients, r < 0.5), and biologically plausible predictors of the outcome of interest were considered (Additional files 1, 2, 3). This was followed by a stepwise, backward removal of non-significant variables with the highest *P* value. Parsimonious, final models contained only predictor variable(s) for which *P* < 0.05. All final models were evaluated to ensure linearity, normality and homoscedasticity of residuals.

To determine if the number of dead adjacent fetuses differed across preservation category, a two-level Poisson regression model (MEPOISSON) accounting for dam of origin as a random effect was developed. Similarly, MEPOISSON was used to determine if the number of adjacent PRRSV RNA positive fetuses differed between PRRSV RNA positive and negative fetuses.

#### Objective 1

A two-level linear, mixed-effects regression model (XTMIXED) was used to identify potential gilt level factors, measured before inoculation, associated with fetal mortality rate (Additional file 4). The model included the sire of the litter as a random effect. The predictive ability of this model was assessed by plotting the predicted outcome for each animal against the raw data in order to visually assess their agreement. Then, a simple linear regression was performed to determine the strength of the association between the predicted and raw values.

#### Objective 2

A three-level, mixed-effects logistic regression model (XTMELOGIT) was used to assess potential factors associated with odds of fetal death. The model included the sire and dam of the litter as random effects. Because model diagnostics indicated a non-linear relationship between the outcome and PRRS viral load at the maternal-fetal interface, a quadratic term was included in the full and final models (Additional file 5). The predictive ability of this model was assessed by generating post-estimation classification statistics and receiver operating characteristic (ROC) curves as described by Dohoo et al. [25], based on a cut off probability of 0.5 (e.g. fetus was classified as live if it had predicted probability of death <0.5; dead fetuses >0.5).

#### Objective 3

A three-level, mixed-effects linear regression model (XTMIXED) was used to assess potential factors associated with PRRS viral load in fetal thymus (Additional file 6). The model included the sire and dam of the litter as random effects. The predictive ability of this model was assessed as described for objective 1.

To graphically illustrate the relative influence of each variable retained in each of these three final models (objectives 1–3), predictive margin plots (MARGINSPLOT) were developed whereby each factor, across its natural range, was plotted against the appropriate outcome (percent dead fetuses, odds of fetal death, fetal thymic viral load) while keeping the other retained factors constant at their mean. The natural range selected for all three models encompassed the 5th to the 95th percentile values for each variable.

#### Objective 4

Having identified the factors significantly associated with fetal death and fetal thymic viral load, the intent of this final objective was to extend this analysis to determine which of those factors were predictive of fetal preservation category and elucidate the relative importance of events occurring in the maternal and fetal compartments. A two-level partial proportional odds model (GLLAMM), accounting for the dam as a random effect, was used. The full model included only the factors found to be significantly associated with the odds of fetal death or fetal viral load (Additional file 7). A final, parsimonious model was achieved through backward, stepwise elimination of variables with the highest *P* value. In the full and final models, it was necessary to constrain some of the variables in order to ensure the proportional-odds assumption was not violated. The predictive ability of the five significant factors retained in the final model was assessed using linear discriminant analysis (CANDISC) using the proportional group-size “priors option” to reflect that fetuses were not equally distributed across all four fetal preservation categories. A “leave-one-out table” was generated to determine the percentage of fetuses correctly categorized in each fetal preservation category.

## Results

### Phenotypic outcomes of PRRSV infection measured in gilts and fetuses

Detailed results on clinical signs, viral loads (serum and tissues) and litter outcomes including fetal preservation can be found in Ladinig et al. [19]. Briefly, all inoculated gilts were viremic at 2 and 6 dpi, and 94/111 (84.7%) remained viremic until termination. Tissue samples from most challenged gilts tested positive by PRRSV qRT-PCR at termination (21 dpi). Eighty-five percent of fetal-maternal interface (1185/1392 samples; mean 3.9 ± 1.7 log_10_ copies/mg) and 72.8% of fetal thymus samples (1013/1391 samples; mean 4.7 ± 2.0 log_10_ copies/mg) also tested positive by qRT-PCR. The mean litter size excluding MUM was 12.5 ± 3.7. The fetal mortality rate per litter ranged from 0% to 94.4% (mean 41.0 ± 22.8%), with 50% of fetuses categorized as VIA, 9% as MEC, 8% as DEC, and 33% as AUT [19]. Dead fetuses clustered within the uterus and the number of adjacent dead fetuses was significantly lower for VIA fetuses compared to all other preservation categories (VIA < MEC by 0.29; VIA < DEC by 0.26; VIA < AUT by 0.37; *P* < 0.01 for all). PRRSV RNA positive fetuses also clustered within the uterus. PRRSV RNA positive fetuses had on average 0.4 more positive adjacent neighbors (*P* < 0.001) than did PRRSV RNA negative fetuses.

Detailed results of cytokine responses after PRRSV inoculation are presented in Ladinig et al. [18]. A massive decrease in total WBC was detected at 2 dpi, which was correlated with a similar decrease in all investigated PBMC subsets to varying degrees, and most severely in NK cells and CTLs. All PBMC subsets, except B cells, started to rebound by 6 dpi [17].

Due to the lack of consistent and severe histologic lesions in most of the gilt and fetal tissues, only the presence or absence of lesions in myometrium and fetal placenta were included in the statistical analyses. Characteristic lesions of lymphohistiocytic infiltration and vasculitis were observed in the endometrium adjacent to all except for four fetuses. There was also lymphohistiocytic placentitis with occasional vasculitis for 83/1309 fetuses and mild lymphocytic myometritis with accompanying lymphocytic perivascular cuffing for 765/1309 fetuses.

### WUR10000125 allele of gilts, sires and fetuses

Seventy-nine of 114 gilts (69%) were homozygous AA, 30/114 (26%) heterozygous AG, and 2/114 gilts homozygous GG. With the exception of one boar, which had an AG genotype, all boars used to breed the gilts were AA. The WUR10000125 allele could be determined in 928 non-autolyzed fetuses: 761 fetuses were AA, 160 were AG, and 7 fetuses had GG genotype.

### Gilt level factors of fetal mortality rate (objective 1)

**Figure 1:**
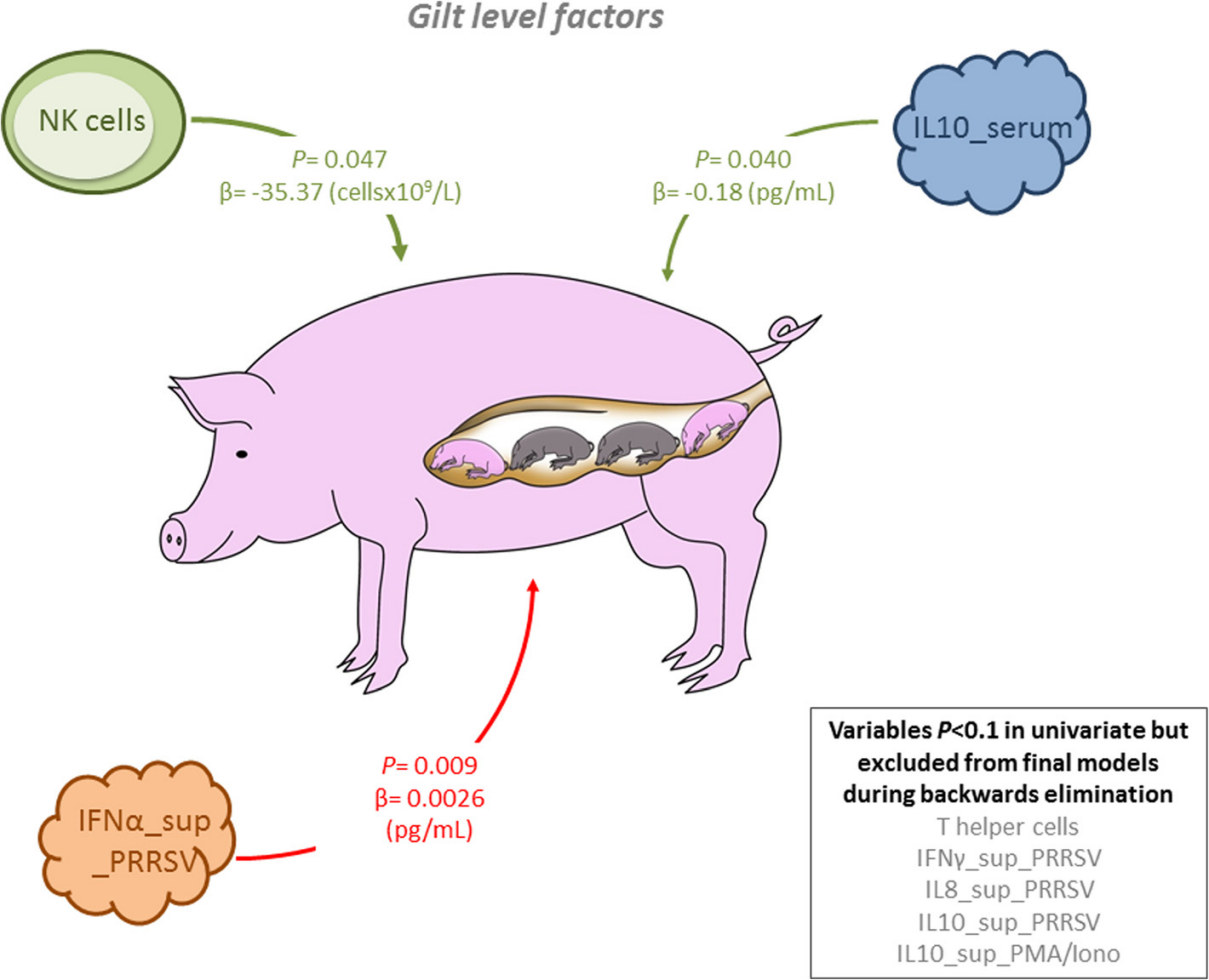
**Gilt level predictors, measured before inoculation, associated with the fetal mortality rate.** Grey font (text box at bottom right) indicates variables that were included in the full model (*P* < 0.1 in unconditional analyses), but were excluded from final models due to non-significance during stepwise backwards elimination. Levels of IFNα in supernatants of PRRSV stimulated PBMC (IFNα_sup_PRRSV; pg/mL) were positively associated (red arrow) with fetal mortality rate, whereas both IL10 in serum (IL10_serum; pg/mL) and absolute numbers of NK cells (cells × 10^9^/L) were negatively associated (green arrows) with fetal mortality rate. *P*-values and coefficients (β) are indicated for significant factors.

**Figure 2:**
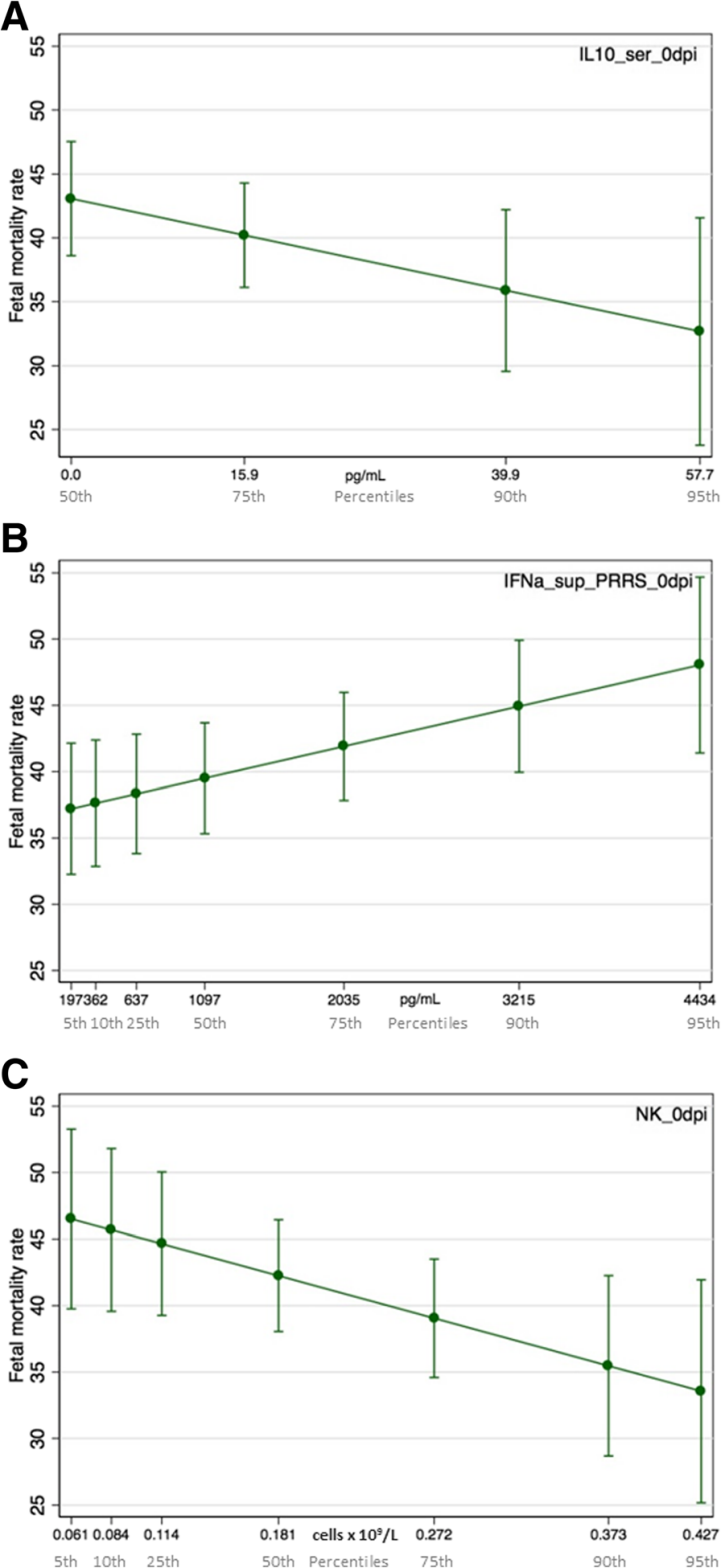
**Margin plots showing relative influence of statistically significant gilt level factors on fetal mortality rate.** The predicted effect on fetal mortality rate was calculated across the natural range of X-axis levels while all other covariates were kept constant at their mean. The slopes of the lines show that IFNα levels were positively associated with fetal mortality rate (**b**) while IL10 (**a**) and NK cells (**c**) were negatively associated with the fetal mortality rate. Error bars represent 95% confidence intervals.

Eight gilt-level variables, measured before inoculation, were included in the full model investigating the factors associated with fetal mortality rate (Additional file 4). During stepwise, backward elimination, five variables were removed as non-significant, leaving three significant associations (Figure 1). IFNα levels were positively associated with fetal mortality rate, whereas both IL10 and NK cells were negatively associated. Across their natural range of values, the three significant factors in the final model had similar influence on fetal mortality rate (Figure 2). The final model, however, had poor predictive ability (adjusted R^2^ = 0.04; Additional file 1) raising doubts about its utility.

### Gilt and fetal level factors associated with odds of fetal death (objective 2)

**Figure 3:**
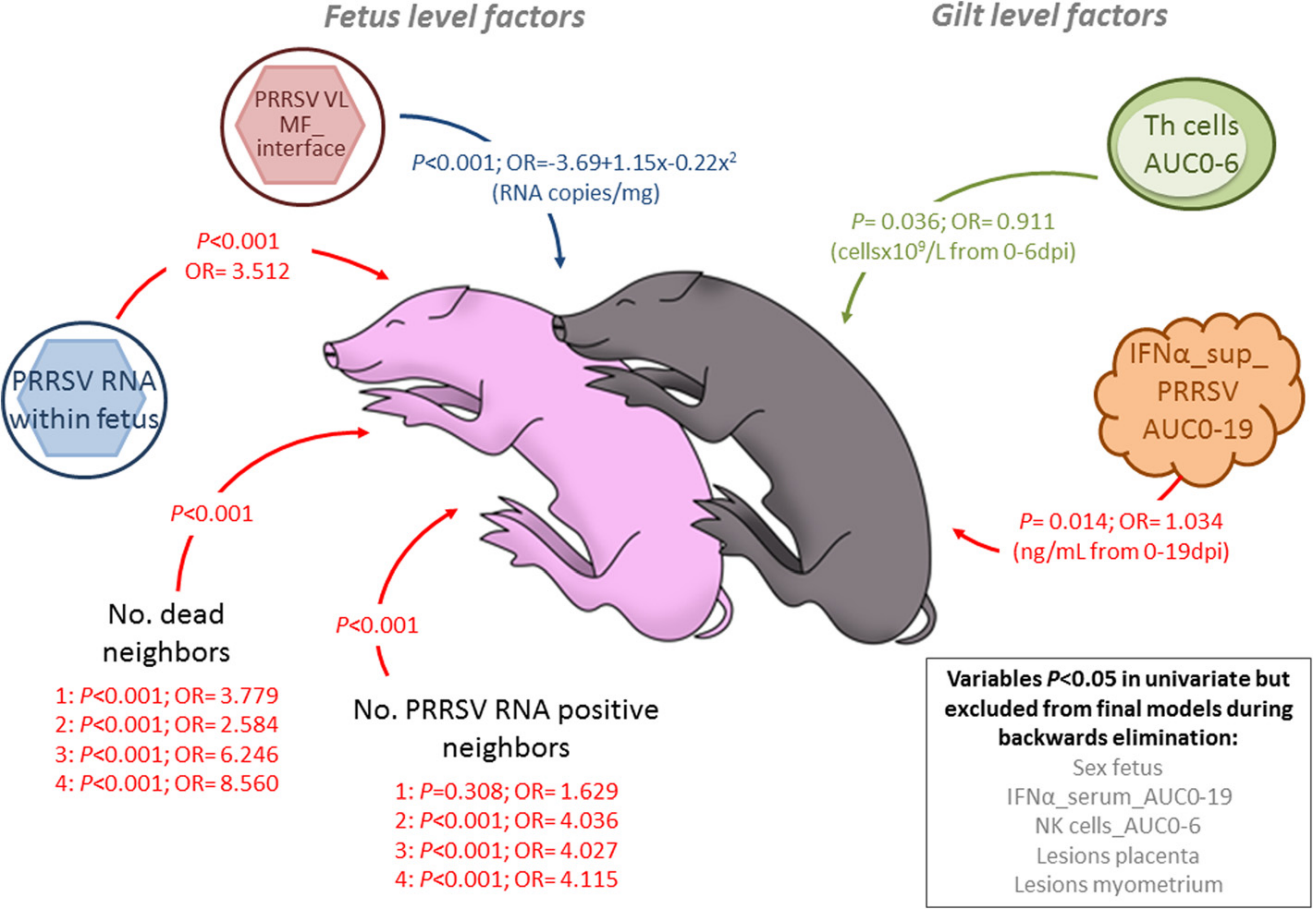
**Gilt and fetal level predictors of the odds of fetal death.** Grey font (text box at bottom right) indicates variables that were included in the full model (*P* < 0.05 in unconditional analyses), but were excluded from final models due to non-significance during stepwise backwards elimination. Levels of IFNα in supernatants of PRRSV stimulated PBMC over time (IFNα_sup_PRRSV AUC0-19) increased the odds of fetal death (red arrows), while absolute numbers of T helper lymphocytes early after infection (Th cells AUC0-6) were protective (green arrow). The presence of PRRSV RNA (PRRSV RNA within fetus), adjacent PRRSV RNA positive fetuses (No. PRRSV RNA positive neighbors), and adjacent dead fetuses (No. dead neighbors) significantly increased the odds of fetal death. Assessment of PRRSV RNA concentration measured at the maternal-fetal interface (PRRSV VL MF_interface) required the inclusion of a quadratic term in the statistical model (blue arrow). Low viral load at the MF interface was associated with increasing odds of fetal death, while high viral load was associated with decreasing odds of fetal death. *P*-values and odds ratios are indicated for all significant factors.

**Figure 4:**
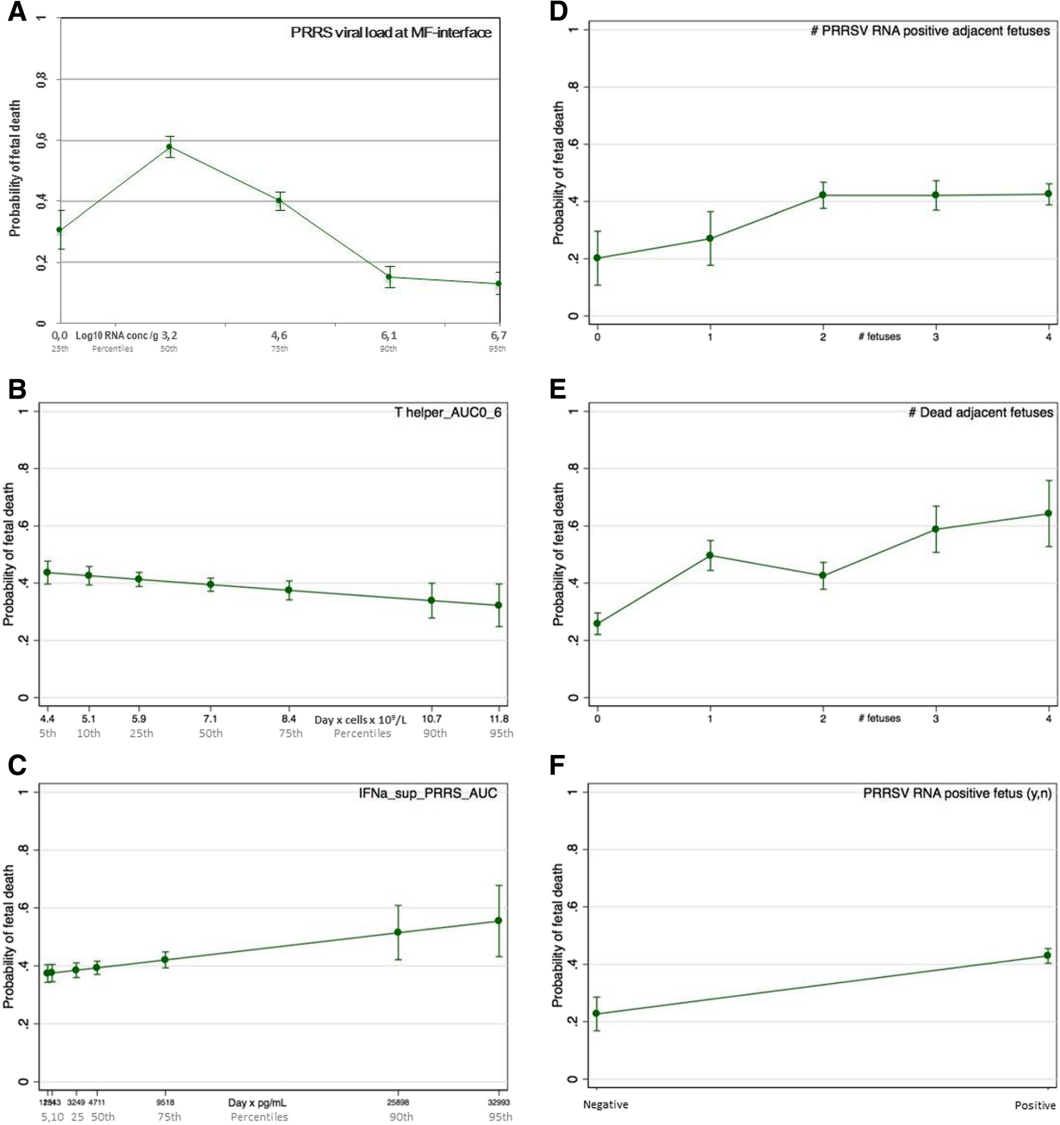
**Margin plots showing relative influence of significant factors on probability of fetal death.** The probability of fetal death was calculated across a natural range of X-axis levels while the remaining factors were kept constant at their mean. The slopes of the lines demonstrate that high INFα levels contributed to fetal death (**C**), while elevated T helper cell numbers were protective (**B**). PRRSV RNA concentration measured at the maternal-fetal interface (**A**) had a quadratic relationship with fetal death probability. Additional adjacent PRRSV RNA positive and dead fetuses also contributed to probability of fetal death (**D**, **E**), as did the presence of PRRSV RNA in the fetus (**F**). Error bars represent 95% confidence intervals.

Four gilt-level and seven fetal-level factors were included in the full model investigating factors associated with the odds of fetal death (Additional file 5). For these analyses, AUC0-6 or AUC0-19 were included as appropriate, because they reflected a greater post-inoculation period than cytokine levels or PBMC counts on any individual day. During stepwise, backward elimination, five variables were removed due to non-significance, leaving six significant associations. Two gilt-level factors were significantly associated with fetal death (Figure 3): levels of IFNα in supernatants of PRRSV stimulated PBMC (AUC0-19) and absolute numbers of T helper cells (AUC0-6). Whereas high INFα levels contributed to fetal death, T helper cells were potentially protective. In addition, four fetal level factors were significantly associated with fetal death (Figure 3). The presence of detectable PRRSV RNA in a fetus, and each additional adjacent PRRSV RNA positive or adjacent dead fetus increased the odds of fetal death. PRRSV RNA concentration at the maternal-fetal interface had a dose dependent effect. Concentrations up to about 3 logarithm (base 10) per gram were associated with increasing probability of fetal death, whereas RNA concentration over 3 logarithm (base 10) were associated with decreasing probability of fetal death (Figure 4A). Across their natural range, changes in T helper cell numbers (Figure 4B) had a small protective effect, while changes in IFNα (Figure 4C) had a contributory effect on fetal death probability. The substantial impact of adjacent fetuses was clearly apparent; the probability of fetal death doubled from 0.2 to 0.42 as the number of adjacent PRRSV RNA positive fetuses increased from 0 to 2 (Figure 4D) and more than doubled from 0.26 to 0.64 as the number of adjacent dead fetuses increased from 0 to 4 (Figure 4E). The probability of fetal death increased from 0.23 to 0.43 if the fetus was PRRSV positive (Figure 4F). The final model had moderate predictability (area under ROC curve = 0.83) and correctly classified 76% of fetuses as live or dead. As demonstrated by the ROC curves (Additional file 2), the model was superior at predicting live than dead fetuses and would have equivalent sensitivity and specificity at a probability cutoff of about 0.45 (fetuses classified as dead if predicted probability >0.45).

### Gilt and fetal level factors associated with PRRS viral load in fetal thymus (objective 3)

**Figure 5:**
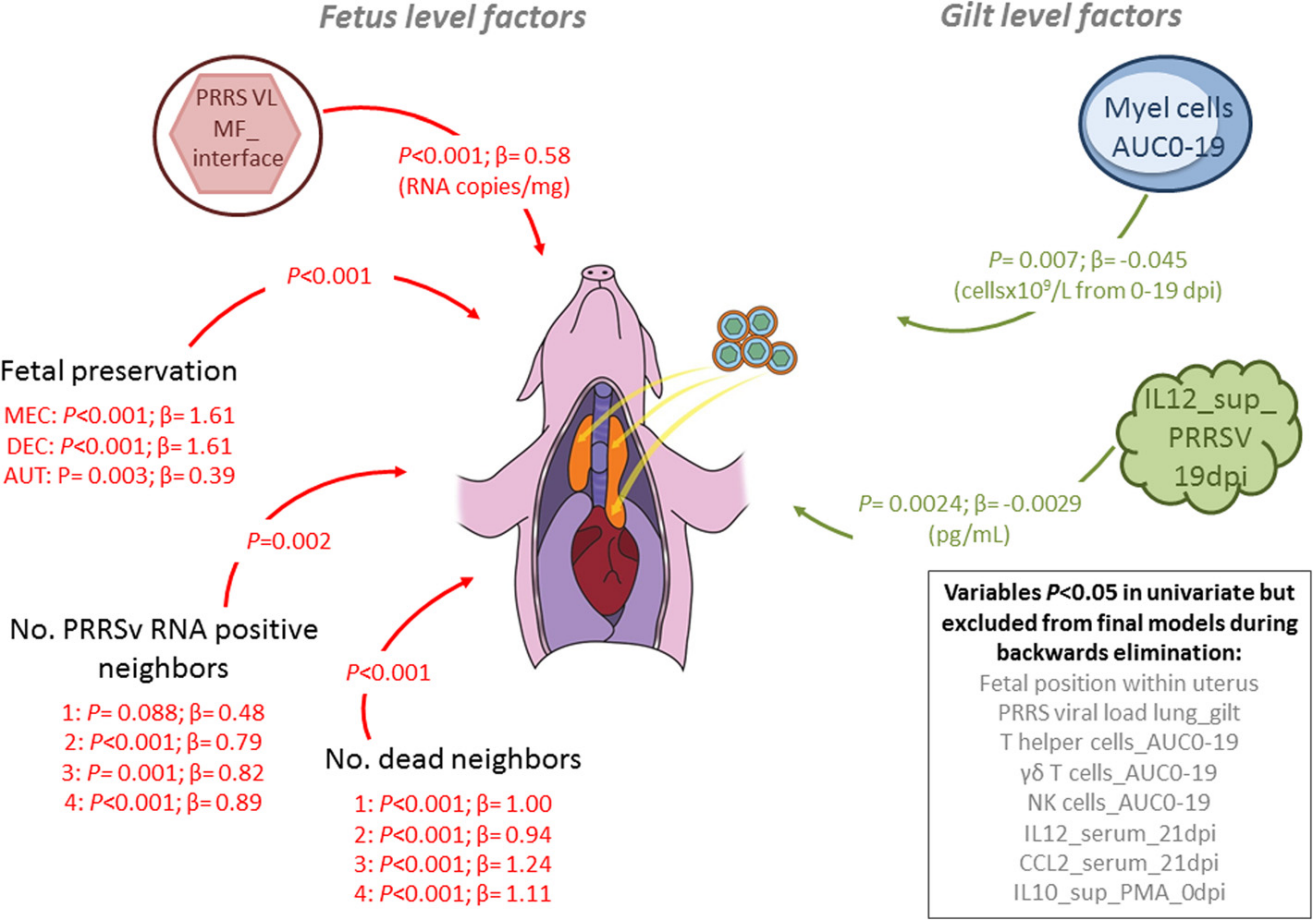
**Gilt and fetal level predictors of viral load in fetal thymus.** Grey font (text box at bottom right) indicates variables included in the full model (*P* < 0.05 in unconditional analyses), but excluded from final models due to non-significance during stepwise, backwards elimination. Levels of IL12 in supernatants of PRRSV stimulated PBMC collected at 19 dpi (IL12_sup_PRRSV_19 dpi) and absolute numbers of myeloid cells over time (Myel cells AUC0-19) were negatively associated (green arrows) with viral load in fetal thymus. Preservation category (viable (VIA), meconium stained (MEC), decomposed (DEC), autolyzed (AUT)), viral load at the maternal-fetal interface (PRRS VL MF_interface), the number of adjacent dead fetuses (No. dead neighbors) and the number of adjacent PRRSV RNA positive fetuses (No. PRRSV RNA positive neighbors) were positively associated (red arrows) with thymic viral load. *P*-values and coefficients (β) are indicated for significant factors.

**Figure 6:**
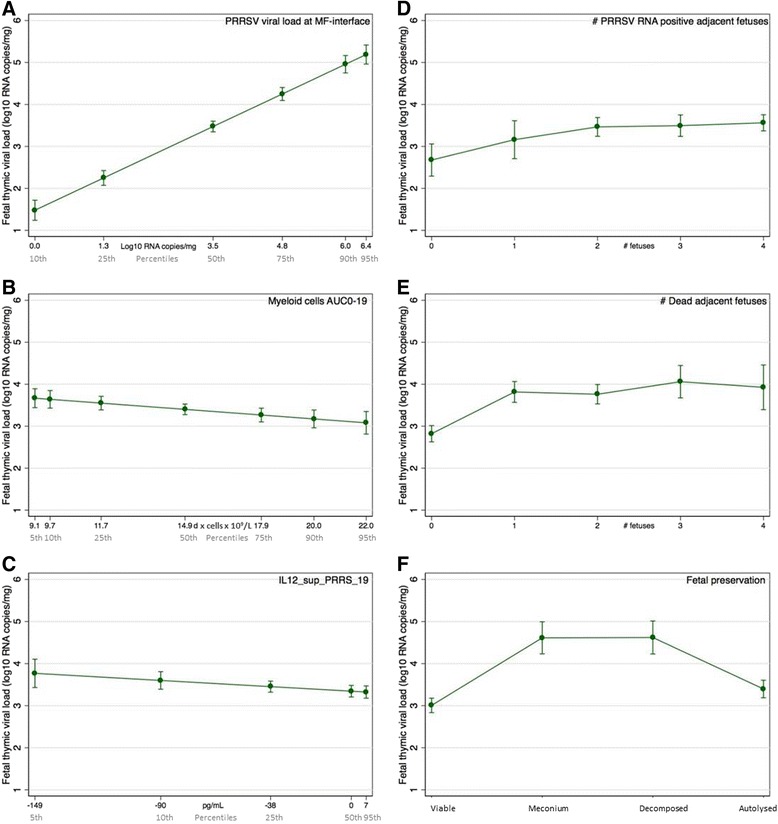
**Margin plots showing relative influence of statistically significant factors on PRRSV load in fetal thymus.** The predicted effect on viral load in fetal thymus was calculated across a natural range of X-axis levels while the remaining factors were kept constant at their mean. Levels of IL12 (**C**) in supernatants of PRRSV stimulated PBMC collected at 19 dpi and absolute numbers of myeloid cells over time (AUC0-19; **B**) were negatively associated with viral load in fetal thymus. Fetal preservation category (**F**), viral load at the maternal-fetal interface (**A**), the number of adjacent dead fetuses (**E**), and the number of adjacent PRRSV RNA positive fetuses (**D**) were positively associated with thymic viral load. Of the six factors, PRRS RNA concentration at the maternal-fetal interface clearly demonstrated the largest effect on fetal viral load in this experiment. Error bars represent 95% confidence intervals.

Nine gilt-level and five fetal-level variables were included in the full model investigating potential factors of viral load in fetal thymus (Additional file 6). PBMC counts were represented by AUC0-19, but because no cytokine AUCs were significant in the unconditional analyses, cytokine levels on individual days were included in the model as appropriate. Eight factors were removed during stepwise backwards elimination, leaving six significant associations. Two gilt-level factors were negatively associated with viral load in fetal thymus (Figure 5): levels of IL12 in supernatants of PRRSV stimulated PBMC collected at 19 dpi and absolute numbers of myeloid cells over time (AUC0-19). Four fetal-level factors were positively associated with thymic viral load including fetal preservation category, viral load at the maternal-fetal interface, the number of adjacent dead fetuses and the number of adjacent PRRSV RNA positive fetuses. Of these six factors, PRRSV RNA concentration at the maternal-fetal interface clearly demonstrated the largest effect on thymic viral load (Figure 6A). By contrast myeloid cell numbers (Figure 6B) and IL12 (Figure 6C) had only a modest influence on fetal thymic load, although both were protective. The number of adjacent PRRSV RNA positive (Figure 6D) or dead (Figure 6E) fetuses also influenced thymic viral load, but most of the influence was associated with the first two adjacent fetuses, suggesting inter-fetal transmission may occur. Thymic viral load was greatest in MEC and DEC fetuses (Figure 6F) indicative of active viral replication. Viral load was lower in VIA fetuses as expected, and in AUT fetuses presumably due to post-mortem viral RNA degradation over time. The final model had moderate predictive ability (adjusted R^2^ = 0.50; Additional file 3) except for fetuses with no detectable PRRSV RNA in fetal thymus.

### Gilt and fetal level factors associated with fetal preservation category (objective 4)

**Figure 7:**
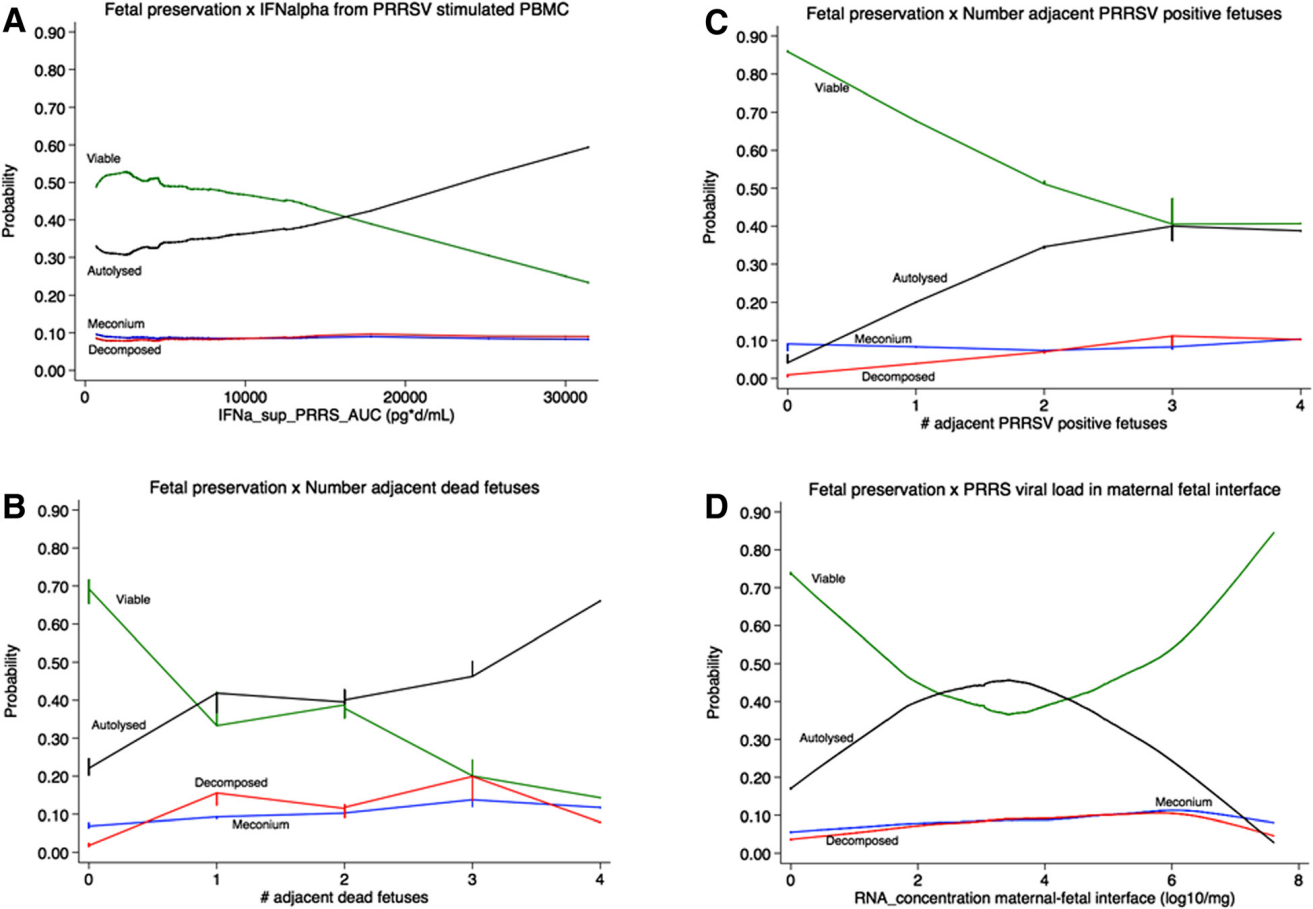
**Maternal and fetal predictors of fetal preservation category.** Results of multi-level, partial proportional odds regression model showing the predicted probability of fetuses being viable (VIA; green line), meconium stained (MEC: blue line), decomposed (DEC: red line) or autolyzed (AUT; black line) based on: **A** levels of interferon alpha (IFNalpha) produced by PRRSV stimulated peripheral blood mononuclear cells (PBMC); the number of **B** adjacent dead or **C** adjacent PRRSV positive fetuses; and **D** the concentration of PRRSV RNA in the maternal fetal interface. The greatest probability of a viable fetus is associated with low viral load in the maternal fetal interface, low interferon alpha levels and no adjacent dead or PRRSV positive fetuses. The greatest probability of an autolyzed fetus is associated with mid-range viral load in the maternal fetal interface, high interferon alpha levels, four adjacent dead fetuses and more than two adjacent PRRSV positive fetuses.

Of the eight variables included in the full model (significant factors from objectives 2 and 3), one gilt-level and four fetal-level variables were retained in the final model as significant predictors of fetal preservation category (Additional file 7). The presence of PRRSV RNA in fetal serum or thymus significantly decreased the probability of a fetus being VIA, while increasing the probability of being MEC, DEC or AUT. Increasing levels of IFNα in supernatants of PRRSV stimulated PBMC over time (AUC0-19) were associated with increased probability of AUT and decreased probability of VIA fetuses (Figure 7A). Similarly, increasing numbers of adjacent dead and PRRS RNA positive fetuses increased the probability of AUT and decreased the probability of VIA fetuses (Figure 7B and C). Viral load at the maternal-fetal interface had a dramatic effect on the probability of fetuses being VIA or AUT (Figure 7D). While the probability of being an autolysed fetus was highest at mid VL levels, the highest likelihood of VIA was seen with very high and very low VL levels. Results of the linear discriminant analysis indicated that these five significant factors had moderate predictive ability, and correctly classified the preservation category of 63% of fetuses on average (VIA 72%, MEC 60%, AUT 65%). However, these five factors were unable to predict which fetuses were DEC.

## Discussion

This study summarizes the analyses of an extensive and complex dataset of broadly characterized phenotypic responses in third trimester pregnant gilts and their fetuses following experimental type 2 PRRSV infection with the overarching goal to elucidate gilt and fetal factors associated with reproductive pathophysiology.

Due to the large number of factors evaluated we were conservative in building the multi-level regression models by only including in the full models, biologically plausible predictor variables of the outcome of interest if *P* < 0.05 or *P* < 0.1 in unconditional analyses. In addition, the predictive ability of each final model was assessed to ensure the statistical analyses were valid. Unfortunately, the predictive ability of the gilt-level fetal mortality model was unsatisfactory. This suggests that reproductive outcome cannot be accurately predicted based on pre-inoculation testing of gilt immunologic phenotypes, so this analysis will be discussed no further.

It is clear from the three fetal level statistical models (objectives 2, 3, 4) that fetal outcome is dependent on events occurring in the fetal compartment as well as at the maternal-fetal interface. A strong predictor of fetal thymic viral load, based on our extensive dataset, is the concentration of PRRSV RNA at the maternal fetal interface. This variable was also associated, in a curvilinear manner, with the probability of fetal death, fetal viability, and fetal autolysis. Although a curvilinear relationship may seem contradictory, it is likely that VIA fetuses with low VL were the most resistant in the litter, whereas VIA fetuses with high VL were recently infected and actively replicating PRRSV; many of which would die within a short period of time, before the concentration of PRRSV RNA begins to decline associated with tissue autolysis. Although RNA concentration at the maternal-fetal interface was clearly important, the pathophysiologic mechanism of the virus is not fully understood. Karniychuk et al. [2,10,23] proposed that PRRSV crosses the placenta in permissive maternal macrophages, then replicates in fetal macrophages causing focal apoptosis and detachment of the fetal allantochorion from the uterine epithelium. Although our sampling technique was insufficient to distinguish viral levels in the endometrium and allantochorion, our results support the importance of virus levels in the maternal-fetal interface. By contrast, viral loads in gilt serum and systemic and lymphoid tissues were not associated with fetal outcome indicating that the concentration of PRRSV in non-reproductive systemic or lymphoid tissues is of little importance in the reproductive model, except that it may “seed” the maternal-fetal interface.

The pathogenesis of fetal death, however, is complex and clearly associated with more than viral load at the maternal-fetal interface. Our results are consistent in that the presence of PRRSV RNA within fetuses and the status of adjacent fetuses were strongly associated with thymic viral load and the probability of fetal death. The importance of fetal infection as a prerequisite to fetal death is supported by our previous report that over 90% of DEC and AUT fetuses were PRRSV RNA positive [19] compared to 60% in VIA fetuses. Interestingly, the margin plots reported herein (Figures 4 and 6) indicate that the first two adjacent fetuses generally exert the greatest influence. This concept of “influential adjacent fetuses” explains why both dead fetuses and PRRSV RNA positive fetuses cluster within the uterus. These findings indicate that PRRSV is likely transmitted between adjacent fetuses, as previously reported in the transmission of porcine parvoviruses and porcine circovirus type 2 [26,27]. The demonstration of microchimerism in late term porcine fetuses also supports this theory and indicates a potential mechanism of inter-fetal PRRSV transmission [28].

DEC and AUT fetuses, while both dead, were arbitrarily differentiated based on the percentage of their skin that was brown versus white at necropsy. The goal of differentiating these preservation categories was to help identify the population of fetuses (AUT) that were most susceptible to PRRSV infection. It is most likely that the arbitrary nature of these categories is why the partial proportional odds model (objective 4) failed to satisfactorily predict the probability of DEC fetuses. In spite of this, the same combination of fetal and maternal factors correctly predicted the preservation category of over 60% of VIA, MEC and DEC fetuses. While this model provides additional evidence of the importance of these factors in the pathophysiology of fetal outcome, it indicates that there are other undefined maternal or fetal factors that are also important. We have ongoing analyses investigating the role of host genomics and anticipate that they may help to elucidate this issue. It is noteworthy, however, that the deleterious nature of IFNα on fetuses was consistent in models of both fetal death and fetal preservation category.

Even though viral load in the dam’s serum and non-uterine tissues were not associated with fetal outcome, a number of gilt-level factors were important predictors of fetal death probability and viral load in thymus. Of the six PBMC subsets measured in gilt blood, only two were associated with reproductive outcome. Absolute numbers of myeloid cells measured over 19 dpi and T helper cells measured in the early post-inoculation period were negatively associated with viral load in fetal thymus and fetal death, respectively. These results suggest that these subsets may help protect against PRRS severity, and potentially virus transmission from the dam to her fetuses. Myeloid cells are of particular importance in regards to PRRSV infection, since porcine alveolar macrophages are the primary target cells of virus replication [29,30]. Furthermore, it has been suggested that the number of Sn^+^/CD163^+^ macrophages in the maternal-fetal interface might be essential for transplacental virus passage [11]. The only other report investigating changes in absolute numbers of myeloid cells in peripheral blood of pregnant sows found that a significant decrease occurred in inoculated animals at 3 to 7 dpi compared to non-infected controls. Myeloid cell counts returned to control levels by 14 dpi [5]. CD4^+^ T cells, or T helper cells, exhibit diverse functions crucial for an adaptive immune response. After activation through pathogen peptides presented by major histocompatibility complex (MHC) class II molecules, naïve T helper cells can differentiate into different effector subsets which can either have activating or regulating functions [31]. We detected a severe drop in total T helper cell counts early after PRRSV infection in our pregnant gilt model [17] and the multi-level regression model suggested that a more severe drop in the early post-inoculation period, resulting in lower AUC0-6 values, might have negative effects on the outcome of infection. Further studies are required to confirm the association of T helper cells with severity of reproductive PRRS.

Of all eight investigated cytokines, IFNα was the only cytokine associated with fetal death probability. IFNα is involved in the defense of viral infections by promoting resistance to viral replication, MHC class I expression, and activation of NK cells [31]. Although IFNα is a potent antiviral molecule, our results demonstrate that it has negative effects in reproductive PRRSV infection, which is supported by the knowledge that IFNα up-regulated the expression of sialoadhesin and therefore enhanced PRRSV infection of monocytes [32]. It is therefore plausible that IFNα indirectly enhanced transplacental passage of PRRSV, leading to fetal infection. Alternately, the higher levels of IFNα might indicate more sustained expression of IFNα in susceptible gilts as they take longer to control PRRSV replication [33]. Levels of IL12 produced by PRRSV stimulated PBMC at 19 dpi were negatively associated with viral load in fetal thymus, thus potentially have a positive influence on reproductive outcome. IL12 plays important roles in innate immune responses through the activation of NK cells, and also in adaptive immune responses through the activation of CD4^+^ T cells to develop into Th1 cells [31].

In contrast to nursery pigs used in a respiratory PRRS model [20-22], in the present experiment the WUR10000125 SNP on SSC4, in both gilts and fetuses, was not associated with fetal death or viral load. Unfortunately, AUT fetuses, which were dead for at least one week prior to termination and were likely most susceptible to PRRSV infection, could not be genotyped since high quality genomic DNA was not isolated. However, a recent study exploring the genetic basis of host response to PRRSV during a PRRS outbreak in a commercial multiplier sow herd also did not find an association between the WUR10000125 SNP and reproductive performance [34]. Thus, results from this present study and the outbreak herd suggest that the SSC4 region does not explain variation in susceptibility in reproductive PRRS. Further experiments are ongoing to confirm this finding.

Based on the present results, there is no evidence that the severity of reproductive PRRS is related to the birth weight of the dam. In humans, birth weight and intrauterine growth retardation were associated with increased risk of infectious disease mortality [35-37], decreased thymic function [38], impaired cell-mediated immunity [39] and impaired humoral immune responses following typhoid vaccination [40,41]. The characterization of immune responses and disease susceptibility in low BW pigs is incomplete and this study provided the first insights in regards to PRRSV infection. Fetal characteristics including sex, CRL, the position of the fetus within the uterine horns were also not related to fetal death or viral load in fetal thymus, indicating that these factors do not play a role in the pathogenesis of fetal infection and death.

Due to the fact that gilts were inoculated with one strategically chosen type 2 PRRSV isolate, results of the present experiment should be extrapolated to other PRRRSV strains with some caution. Follow up experiments using type 1 and other type 2 PRRSV strains are needed to confirm the results presented herein. That being said, as a prequel to this challenge experiment, two other type 2 PRRSV strains were evaluated in this pregnant gilt model with similar phenotypic results [15]. However, insufficient animal numbers prevented their inclusion in the present analyses. Because all animals were terminated 21 days post inoculation, a time when about one third of the fetuses had been dead for greater than 1 week, early phenotypic responses associated with the death of the most susceptible fetuses were not evaluated in the present study. Therefore, we plan a follow-up experiment with different termination time points in order to investigate key events associated with risk of early fetal infection and death.

In conclusion, our analyses provide a number of novel findings that improve our understanding of the pathophysiology of reproductive PRRS following type 2 infection in third trimester pregnant gilts. Firstly, the WUR10000125 SNP on SSC4, associated with lower PRRS viral load and higher average daily gain in experimentally infected nursery pigs, was not associated with reproductive outcome after PRRSV infection. Similarly, the birth weight of the dam and levels of PRRSV RNA in dam sera and lymphoid tissue did not influence the reproductive outcome. Secondly, PRRSV RNA concentration in the maternal-fetal interface was a strong predictor of fetal viral load and the probability of fetal death, emphasizing its importance in the transmission of the virus from the maternal to the fetal compartment. Thirdly, the presence of PRRSV in fetuses, particularly at high levels in thymus, increased the likelihood of fetal death indicating fetal infection plays a central role. Fourthly, fetal infection and death clustered within the uterus indicating that the status of adjacent fetuses and inter-fetal transmission of PRRSV significantly influence fetal outcome. Lastly, several systemic immune responses measured in gilts also contribute to fetal outcome in a positive or negative manner. Enhanced IFNα response may negatively contribute to fetal death, whereas an enhanced IL12 response and increased myeloid and T helper cell numbers in the blood of the dam may be protective. Taken together, these results provide clear evidence, for the first time, that events occurring in fetuses are essential in the pathogenesis of reproductive PRRS. This has tremendous implications for PRRSV control strategies, and potentially, other viral reproductive diseases of all litter bearing species. Further experiments are required to determine if similar relationships exist with other type 2 and type 1 PRRSV strains.

## Additional files

Additional file 1:
**Predictability of the gilt-level fetal mortality rate linear model.** A scatter plot of actual fetal mortality rates (X-axis) for each of 111 PRRSV-challenged gilts versus values predicted by the final multi-level, linear regression model (Y-axis) demonstrates the model has poor predictability based on the horizontal data trend and the low adjusted regression coefficient (Adj R^2^ = 0.04) of a simple regression of predicted versus actual values. Additional file 2:
**Predictability of the fetal death probability logistic model.** Double receiver operating characteristic (ROC) curves demonstrate the effects of “probability cutoff” on the sensitivity and specificity of the final multi-level logistic regression model. By default, a probability cutoff of 0.5 was used to generate the ROC curves; fetuses with a predicted probability <0.5 were classified as live, whereas fetuses with a predicted probability >0.5 were classified as dead. The sensitivity curve (solid black line) represents the percentage of fetuses that were correctly classified as dead by the model across a range of probability cutoffs. The specificity curve (grey dashed line) represents the percentage of fetuses that were correctly classified as live by the model. At a probability cutoff of 0.5, the model is superior at predicting live fetuses than dead, which implies that factors associated with fetal death, apart from those included in the final model, exist. The contingency table of raw data and summary of model performance are shown in the bottom left and right, respectively. Additional file 3:
**Predictability of the PRRS thymic viral load linear model.** A scatter plot of actual PRRS viral load in fetal thymus (X-axis) for each of 1,302 PRRSV-challenged fetuses versus values predicted by the final multi-level, linear regression model (Y-axis) is shown. Moderate predictability is evident based on the diagonal trend and the adjusted regression coefficient (Adj R^2^ = 0.5) based on a simple regression of predicted versus actual values. Additional file 4:
**Pre-inoculation gilt level factors associated with the fetal mortality rate in type 2 PRRSV inoculated third trimester pregnant gilts.** Biologically plausible variables included in the unconditional, full and final statistical models to investigate factors associated with the fetal mortality rate are listed; all variables were measured in gilts pre-inoculation. Additional file 5:
**Gilt and fetal level factors associated with the odds of fetal death in type 2 PRRSV inoculated third trimester pregnant gilts.** Biologically plausible variables included in the unconditional, full and final statistical models to investigate factors associated with the odds of fetal death are listed; factors were measured at the gilt or fetal level. Additional file 6:
**Gilt and fetal level factors associated with PRRS viral load in fetal thymus in type 2 PRRSV inoculated third trimester pregnant gilts.** Biologically plausible variables included in the unconditional, full and final statistical model to investigate factors associated with PRRS viral load in fetal thymus are listed; factors were measured at the gilt or fetal level. Additional file 7:
**Gilt and fetal level factors associated with fetal preservation category in type 2 PRRSV inoculated third trimester pregnant gilts.** Variables included in the unconditional, full and final partial proportional odds models to investigate factors associated with fetal preservation category are listed; factors were measured at the gilt or fetal level; only factors significantly associated with the odds of fetal death or PRRS viral load in fetal thymus were included.

## Abbreviations

AUC: Area under the curve
AUT: autolyzed
BW: birth weight
CCL2: chemokine ligand 2
CD: cluster of differentiation
CRL: crown rump length
CTLs: cytolytic T lymphocytes
dpi: days post infection
DEC: decomposed
ELISA: enzyme-linked immunosorbent assay
FCM: flow cytometry
FMIA: Fluorescent Microsphere Immunoassays
H&E: Hematoxylin and Eosin
IFN: interferon
IL: interleukin
MHC: major histocompatibility complex
MEC: meconium stained
NK cells: natural killer cells
PBMC: peripheral blood mononuclear cells
PMA/Iono: phorbol myristate acetate/Ionomycin
PBS: phosphate buffered saline
PRRSV: porcine reproductive and respiratory syndrome virus
qRT-PCR: quantitative reverse transcription polymerase chain reaction
QTL: quantitative trait locus
ROC: receiver operating characteristic
Sn: sialoadhesin
SNP: single nucleotide polymorphism
SSC: *Sus scrofa* chromosome
Th1: T helper 1
Th2: T helper 2
VIA: viable
WBCs: white blood cells
