# Supplementary figures and images for: Maternal and fetal predictors of fetal viral load and death in third trimester, type 2 porcine reproductive and respiratory syndrome virus infected pregnant gilts
Source: Vet Res. 2015 Sep 25;46:107. doi: 10.1186/s13567-015-0251-7 (PMC4582889; doi:10.1186/s13567-015-0251-7)

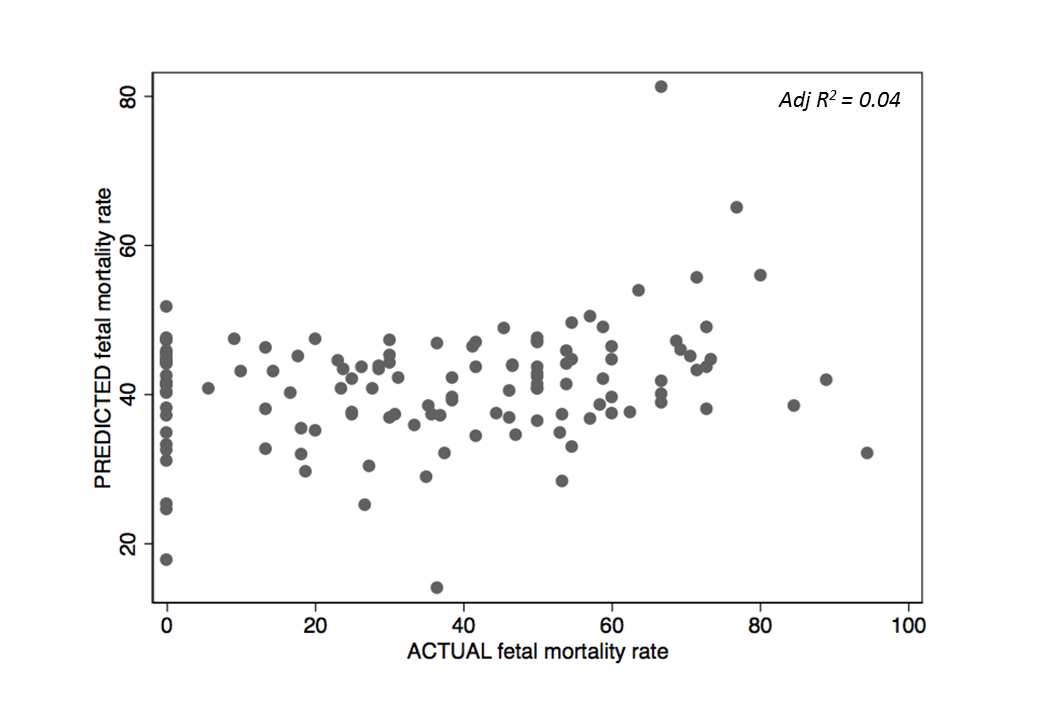

Supplement: Additional file 1: — Predictability of the gilt-level fetal mortality rate linear model. A scatter plot of actual fetal mortality rates (X-axis) for each of 111 PRRSV-challenged gilts versus values predicted by the final multi-level, linear regression model (Y-axis) demonstrates the model has poor predictability based on the horizontal data trend and the low adjusted regression coefficient (Adj R2 = 0.04) of a simple regression of predicted versus actual values. [file 13567_2015_251_MOESM1_ESM.tif]

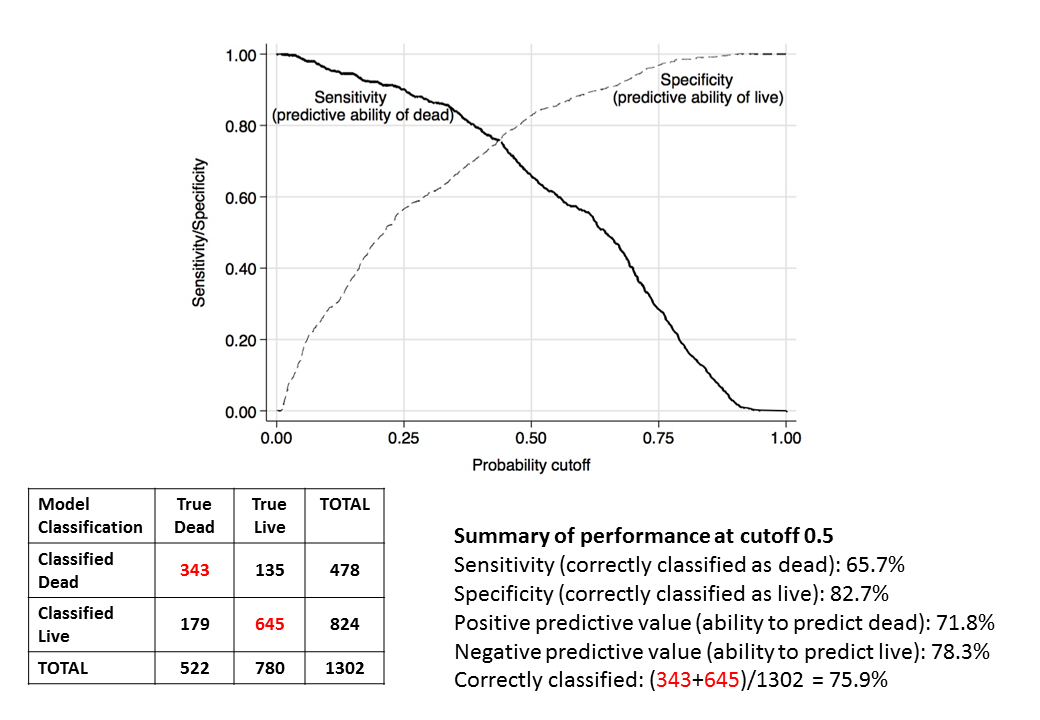

Supplement: Additional file 2: — Predictability of the fetal death probability logistic model. Double receiver operating characteristic (ROC) curves demonstrate the effects of “probability cutoff” on the sensitivity and specificity of the final multi-level logistic regression model. By default, a probability cutoff of 0.5 was used to generate the ROC curves; fetuses with a predicted probability <0.5 were classified as live, whereas fetuses with a predicted probability >0.5 were classified as dead. The sensitivity curve (solid black line) represents the percentage of fetuses that were correctly classified as dead by the model across a range of probability cutoffs. The specificity curve (grey dashed line) represents the percentage of fetuses that were correctly classified as live by the model. At a probability cutoff of 0.5, the model is superior at predicting live fetuses than dead, which implies that factors associated with fetal death, apart from those included in the final model, exist. The contingency table of raw data and summary of model performance are shown in the bottom left and right, respectively. [file 13567_2015_251_MOESM2_ESM.tif]

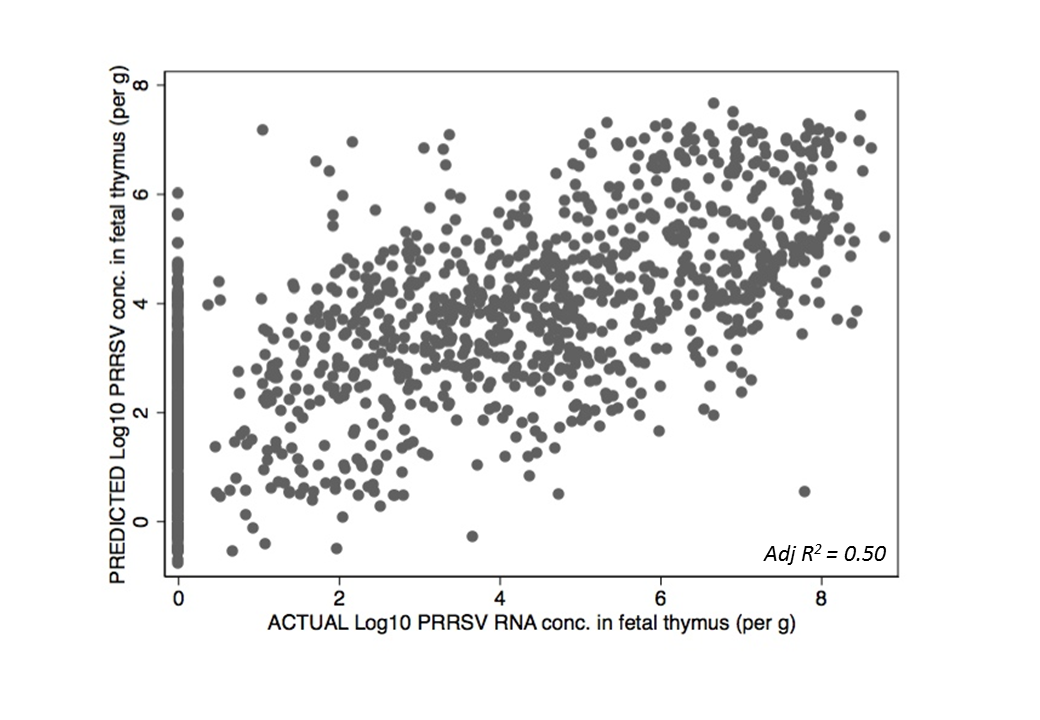

Supplement: Additional file 3: — Predictability of the PRRS thymic viral load linear model. A scatter plot of actual PRRS viral load in fetal thymus (X-axis) for each of 1,302 PRRSV-challenged fetuses versus values predicted by the final multi-level, linear regression model (Y-axis) is shown. Moderate predictability is evident based on the diagonal trend and the adjusted regression coefficient (Adj R2 = 0.5) based on a simple regression of predicted versus actual values. [file 13567_2015_251_MOESM3_ESM.tif]
