# Supplementary material for: Maternal and fetal predictors of fetal viral load and death in third trimester, type 2 porcine reproductive and respiratory syndrome virus infected pregnant gilts
Source: Vet Res. 2015 Sep 25;46:107. doi: 10.1186/s13567-015-0251-7 (PMC4582889; doi:10.1186/s13567-015-0251-7)
Supplement: Additional file 4: — Pre-inoculation gilt level factors associated with the fetal mortality rate in type 2 PRRSV inoculated third trimester pregnant gilts. Biologically plausible variables included in the unconditional, full and final statistical models to investigate factors associated with the fetal mortality rate are listed; all variables were measured in gilts pre-inoculation. [file 13567_2015_251_MOESM4_ESM.docx]

| **Variables included in unconditional analysis** | **Variables included in full model (if *P* < 0.1)** | **Significant in final model (if *P* < 0.05)** | **Effect on fetal mortality rate** |
| --- | --- | --- | --- |
| WUR10000125 allele of gilts (*dichotomous, AA versus AG or GG*) |  |  |  |
| Birth weight status of gilts (*high BW versus low BW*) |  |  |  |
| Cytokine^a^ protein levels in serum at 0 dpi (*pg/mL*) | IL10 serum | IL10 serum | Decreased mortality |
| Cytokine^a^ protein levels in supernatants of PRRSV stimulated PBMC at 0 dpi (*pg/mL*) | IFNα_sup_PRRSV  IFNγ_sup_PRRSV  IL8_sup_PRRSV  IL10_sup_PRRSV | IFNα_sup_PRRSV | Increased mortality |
| Cytokine^a^ protein levels in supernatants of PMA/Iono stimulated PBMC at 0 dpi (*pg/mL*) | IL10_sup_PMA/Iono |  |  |
| Absolute numbers of major PBMC^b^ populations at 0 dpi (*cells x 10^9^/L*) | Th cells  NK cells | NK cells | Decreased mortality |

^a^ Investigated cytokines: IL1β, IL4, IL8, IL10, IL12, CCL2, IFNα, IFNγ.

^b^ Major PBMC populations include: B cells, T cells, T helper (Th) cells, cytotoxic T lymphocytes, natural killer (NK) cells, myeloid cells, gamma delta T lymphocytes (γδ T cells).
