# Supplementary material for: Maternal and fetal predictors of fetal viral load and death in third trimester, type 2 porcine reproductive and respiratory syndrome virus infected pregnant gilts
Source: Vet Res. 2015 Sep 25;46:107. doi: 10.1186/s13567-015-0251-7 (PMC4582889; doi:10.1186/s13567-015-0251-7)
Supplement: Additional file 5: — Gilt and fetal level factors associated with the odds of fetal death in type 2 PRRSV inoculated third trimester pregnant gilts. Biologically plausible variables included in the unconditional, full and final statistical models to investigate factors associated with the odds of fetal death are listed; factors were measured at the gilt or fetal level. [file 13567_2015_251_MOESM5_ESM.docx]

| **Variables included in unconditional analysis** | **Variables included in full model (if *P* < 0.05)** | **Significant in final model (if *P* < 0.05)** | **Effect on odds/probability of fetal death** |
| --- | --- | --- | --- |
| **Gilt level** | | | |
| WUR10000125 allele of gilts (*dichotomous, AA versus AG or GG*) |  |  |  |
| Birth weight status of gilts (*high BW versus low BW*) |  |  |  |
| Litter size (*total number fetuses excluding mummies^a^*) |  |  |  |
| PRRS viral load in serum (*measured on individual days post inoculation and over time as AUC0-21^b,^*^c^) |  |  |  |
| PRRS viral load in gilt tissues^c^ |  |  |  |
| Cytokine^b,d^ protein levels in serum (*pg/mL*) | IFNα_ACU0-19 |  |  |
| Cytokine^bd^ protein levels in supernatants of PRRSV stimulated PBMC (*pg/mL*) | IFNα sup_ PRRSV_AUC0-19 | IFNα sup_ PRRSV_ACU0-19 | Increased odds of fetal death |
| Cytokine^bd^ protein levels in supernatants of PMA/Iono stimulated PBMC (*pg/mL*) |  |  |  |
| Absolute numbers of major PBMC^be^ populations (*cells x 10^9^/L*) | Th_AUC0-6  NK cells_AUC0-6 | Th_AUC0-6 | Decreased odds of fetal death |
| **Fetal level** | | | |
| WUR10000125 allele of fetuses (*dichotomous, AA versus AG or GG*) |  |  |  |
| Sex and crown rump length (*mm*) of fetus | Fetal sex |  |  |
| PRRS viral load at the maternal-fetal (MF) interface^c^ | VL_MF_interface  VL_MF_interface_sq^f^ | VL_MF_interface  VL_MF_interface_sq^f^ | Increased odds of fetal death |
| Presence of detectable PRRSV RNA in the fetus (thymus or serum) | PRRSV RNA positive fetus | PRRSV RNA positive fetus | Increased odds of fetal death |
| Fetal position^g^ |  |  |  |
| Number of dead adjacent fetuses^h^ | No. dead neighbors | No. dead neighbors | Increased odds of fetal death |
| Number of PRRSV RNA positive adjacent fetuses^h^ | No. RNA positive neighbors | No. RNA positive neighbors | Increased odds of fetal death |
| Presence of histologic lesions in the myometrium and fetal placenta | Lesions_placenta  Lesions_myometrium |  |  |

^a^ Mummies (MUM) defined as inspissated fetuses with crown rump length less than 20 cm

^b^ Levels at 0, 2, 6 and 19/21 dpi, AUC0-6 and AUC0-21. AUC = area under the curve. AUC0-21 encompassed 4 time points: 0, 2, 6 and 21 dpi; AUC0-6 encompassed 3 time points: 0, 2 and 6 dpi representing early responses. See Materials and Methods for formula used to calculate AUC.

^c^ PRRSV RNA concentration (log_10_ copies/µL in serum; /mg in tissue).

^d^ Investigated cytokines: IL1β, IL4, IL8, IL10, IL12, CCL2, IFNα, IFNγ.

^e^ Major PBMC populations include: B cells, T cells, T helper (Th) cells, cytotoxic T lymphocytes, natural killer (NK) cells, myeloid cells, gamma delta T lymphocytes (γδ T cells).

^f^ Inclusion of a quadratic term was required to satisfy model assumptions regarding linearity.

^g^ Numbered chronologically; fetal position = fetal number/total fetuses in same horn.

^h^ Range 0 to 4: maximum two on left and two on right.
