# Supplementary material for: Maternal and fetal predictors of fetal viral load and death in third trimester, type 2 porcine reproductive and respiratory syndrome virus infected pregnant gilts
Source: Vet Res. 2015 Sep 25;46:107. doi: 10.1186/s13567-015-0251-7 (PMC4582889; doi:10.1186/s13567-015-0251-7)
Supplement: Additional file 6: — Gilt and fetal level factors associated with PRRS viral load in fetal thymus in type 2 PRRSV inoculated third trimester pregnant gilts. Biologically plausible variables included in the unconditional, full and final statistical model to investigate factors associated with PRRS viral load in fetal thymus are listed; factors were measured at the gilt or fetal level. [file 13567_2015_251_MOESM6_ESM.docx]

| **Variables included in unconditional analysis** | **Variables included in full model (if *P* < 0.05)** | **Significant in final model (if *P* < 0.05)** | **Effect on PRRS viral load in fetal thymus** |
| --- | --- | --- | --- |
| **Gilt level** | | | |
| WUR10000125 allele of gilts (*dichotomous, AA versus AG or GG*) |  |  |  |
| Birth weight status of gilts (*high BW versus low BW*) |  |  |  |
| Litter size (*total number fetuses excluding mummies^a^*) |  |  |  |
| PRRS viral load in serum (*measured on individual days post inoculation and over time as AUC0-21*^b,c^) |  |  |  |
| PRRS viral load (VL) in gilt tissues | Viral load in gilt lung |  |  |
| Cytokine^bd^ protein levels in serum (*pg/mL*) | CCL2_serum_19dpi  IL12_serum_19dpi |  |  |
| Cytokine^bd^ protein levels in supernatants of PRRSV stimulated PBMC (*pg/mL*) | IL12_sup_PRRS_19dpi | IL12_sup_PRRS_19dpi | Decreased fetal viral load |
| Cytokine^bd^ protein levels in supernatants of PMA/Iono stimulated PBMC (*pg/mL*) | IL10_sup_PMA_0dpi |  |  |
| Absolute numbers of major PBMC^be^ populations (*cells x 10^9^/L*) | Myeloid cells_AUC0-19  Th_AUC0-19  γδ T cells_AUC0-19  NK cells_AUC0-19 | Myeloid cells_AUC0-19 | Decreased fetal viral load |
| **Fetal level** | | | |
| WUR10000125 allele of fetuses (*dichotomous, AA versus AG or GG*) |  |  |  |
| Sex and crown rump length (*mm*) of fetus |  |  |  |
| Fetal preservation category | Fetal preservation | Fetal preservation | Increased fetal viral load |
| PRRS viral load at the maternal-fetal interface (MFI)^c^ | VL_MF_interface | VL_MF_interface | Increased fetal viral load |
| Fetal position^f^ | Fetal position |  |  |
| Number of dead adjacent fetuses^g^ | No. dead neighbors | No. dead neighbors | Increased fetal viral load |
| Number of PRRSV RNA positive adjacent fetuses^g^ | No. RNA positive neighbors | No. RNA positive neighbors | Increased fetal viral load |
| Presence of histologic lesions in the myometrium and fetal placenta |  |  |  |

^a^ Mummies (MUM) defined as inspissated fetuses with crown rump length less than 20 cm.

^b^ Levels at 0, 2, 6 and 19/21 dpi, AUC0-6 and AUC0-21. AUC = area under the curve. AUC0-21 of 4 time points: 0, 2, 6 and 21 dpi; AUC0-6 of 3 time points: 0, 2 and 6 dpi representing early responses. See Materials and methods for formula used to calculate ACU.

^c^ PRRSV RNA concentration (log_10_ copies/µL in serum; /mg in tissue).

^d^ Investigated cytokines: IL1β, IL4, IL8, IL10, IL12, CCL2, IFNα, IFNγ.

^e^ Major PBMC populations include: B cells, T cells, T helper (Th) cells, cytotoxic T lymphocytes, natural killer (NK) cells, myeloid cells, gamma delta T lymphocytes (γδ T cells).

^f^ Numbered chronologically, fetal position = fetal number/total fetuses in same horn.

^g^ Range 0 to 4: maximum two on left and two on right.
