# Supplementary material for: Maternal and fetal predictors of fetal viral load and death in third trimester, type 2 porcine reproductive and respiratory syndrome virus infected pregnant gilts
Source: Vet Res. 2015 Sep 25;46:107. doi: 10.1186/s13567-015-0251-7 (PMC4582889; doi:10.1186/s13567-015-0251-7)
Supplement: Additional file 7: — Gilt and fetal level factors associated with fetal preservation category in type 2 PRRSV inoculated third trimester pregnant gilts. Variables included in the unconditional, full and final partial proportional odds models to investigate factors associated with fetal preservation category are listed; factors were measured at the gilt or fetal level; only factors significantly associated with the odds of fetal death or PRRS viral load in fetal thymus were included. [file 13567_2015_251_MOESM7_ESM.docx]

| **Variables included in full model** | | **Significant in final model (if *P* < 0.05)** | |
| --- | --- | --- | --- |
|  | | **Constrained** | **Non-Constrained** |
| **Gilt level** | | | |
|  | IFNα sup_ PRRSV_AUC0-19 | IFNα sup_ PRRSV_AUC0-19 |  |
|  | IL12_sup_PRRS_19dpi |  |  |
|  | Myeloid cells_AUC0-19 |  |  |
|  | T helper_AUC0-6 |  |  |
| **Fetal level** | | | |
|  | VL_MF_interface  VL_MF_interface_sq^a^ | VL_MF_interface  VL_MF_interface_sq^a^ |  |
|  | Presence of detectable PRRSV RNA in the fetus (thymus or serum) |  | Presence of detectable PRRSV RNA in the fetus (thymus or serum) |
|  | No. dead neighbors |  | No. dead neighbors |
|  | No. RNA positive neighbors |  | No. RNA positive neighbors |

^a^ Inclusion of a quadratic term was required to satisfy model assumptions regarding linearity.
